# Pulmonary Complications of Azanucleoside Therapy in Patients with Myelodysplastic Syndrome and Acute Myelogenous Leukemia

**DOI:** 10.1155/2015/357461

**Published:** 2015-12-20

**Authors:** Manuel Molina, Sarvari Yellapragada, Martha Mims, Effie Rahman, Gustavo Rivero

**Affiliations:** ^1^St. Luke's Episcopal Hospital, Houston, TX 77030, USA; ^2^Baylor College of Medicine, Section of Hematology and Oncology, 1 Baylor Plaza, Houston, TX 77030, USA; ^3^The Dan L. Duncan Cancer Center, Baylor College of Medicine, Houston, TX 77030, USA

## Abstract

Our primary aim was to identify potential risk factors and clinical outcome of azanucleoside induced pulmonary complications in patients with myelodysplastic syndrome (MDS) and Acute Myelogenous Leukemia (AML). We present an 89-year-old female with MDS derived AML who developed fatigability, hypoxemia, and bilateral lung infiltrates indicating interstitial lung disease after 11 cycles of azanucleoside. In addition, we describe a cohort of six MDS patients with fever, cough, dyspnea, and pulmonary infiltrates at early time point during azanucleoside treatment. Early and late onset of pulmonary manifestations suggest different pathogenic mechanisms. Brief azanucleoside discontinuation and steroids led to rapid improvement in symptoms.

## 1. Introduction

Myelodysplastic syndrome (MDS) is an immunologically and epigenetically heterogeneous disease characterized by dysplastic hematopoiesis and propensity for AML transformation [1]. Deregulated immunity results in abnormal myelosuppressive cytokine milieu and marrow failure usually observed in low-risk MDS, whereas high-risk disease is associated with high rate of leukemia conversion and expansion of immunosuppressive T cells during progression. Autoimmunity is seen in about 10–20% of patients [2], with pulmonary manifestations representing unusual presentation. Mechanisms associated with autoimmunity and MDS pathogenesis are largely unknown. For patients with high-risk disease, hypomethylating agents such as azacitidine (AZA, Vidaza, Celgene) and decitabine (DAC, Dacogen, Janssen) provide survival benefit. Despite low incidence of side effects, azanucleosides induced lung complications represent a limiting factor for therapeutic optimization. Bronchiolitis obliterans organizing pneumonia (BOOP) and idiopathic pulmonary fibrosis (IPF) are rare noninfectious complications observed in patients treated with azanucleosides who present with fever, dyspnea, and cough. To gain insight into potential clinical, radiographic, and immunological mechanisms of azanucleoside induced lung injury, we present an 89-year-old female who developed IPF after 11 cycles of DAC and we reviewed the English literature on MDS and AML patients who developed noninfectious pulmonary complications while receiving epigenetic therapy.

## 2. Patient and Methods

In addition to our patient, we reviewed 15 MDS cases presenting with BOOP/IPF while on azanucleoside treatment. Cases with incomplete clinical features were excluded resulting in 6 additional patients [3–8]. In our 7 cases' cohort, we evaluated time of symptom onset, age, sex, radiographic and histopathological findings, and cytogenetic and clinical outcome. When available, laboratory data including ANA, P-C ANCA antibodies, erythrocyte sedimentation rate (ESR), and C-reactive protein (CRP) were recorded.

## 3. Case Report

**Figure 1:**
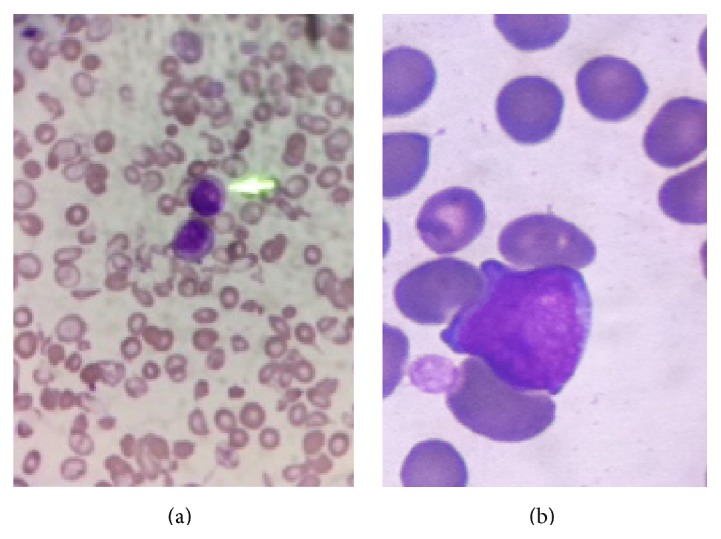
Peripheral smear showing tear drops and fragmented and target red cells. Myeloblasts are shown with yellow arrow (a). Myeloblasts showed high nuclear/cytoplasmatic ratio (b).

**Figure 2:**
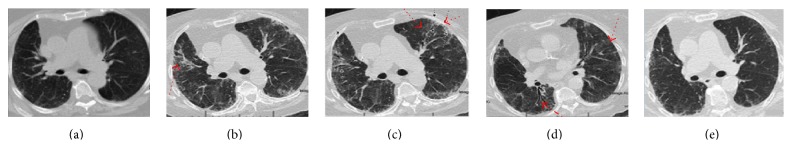
Chest CT at MDS diagnosis (a). Chest CT obtained after 11 cycles of decitabine demonstrating bilateral reticulonodular infiltrates ((b), arrow), subtle early honeycombing (black arrow) in posterior right lung base ((b) and (c), arrow), increased bronchial wall thickening ((d), arrow), and interstitial markings (d). Chest CT after 8 weeks of oral steroids showing progressive resolution of infiltrates (e).

An 89-year-old female presented with pancytopenia. Her WBC was 7800/*μ*L, ANC 1750/*μ*L, absolute lymphocyte count (ALC) 1250/*μ*L, hemoglobin 7.8 g/dL, and platelet count 579000/*μ*L. Peripheral smear showed 15% leukemic blast with high nuclear/cytoplasmatic ratio (Figures 1(a)-1(b)). Her bone marrow demonstrated 50% nucleated cells expressing myeloperoxidase (MPO), CD177, and CD33. Significant erythroid and granulocytic dysplasia suggested AML with myelodysplasia-related changes. Standard G-band karyotyping revealed 54,XX,+1, del(5)(q15q33), +6,+8,+i(11) (q10), +13,+14,+20,+22 [17]/54, idem, del(5q) + del(5) t (5,19) (q15,q13.1) − i(11)(q10), del(19) (q13.1) [3] consistent with complex hyperdiploid karyotype. FISH analysis revealed 5q31, trisomy chromosome 8, deletion 20q, and trisomy 11 in 88%, 85%, 91%, and 10% of nuclei, respectively. Next generation sequencing showed TP53 c.817>C (p.R273G) mutation at exon 8. DAC at 20 mg/m^2^ intravenously for 5 days every 28-day cycle was initiated. A pretreatment chest CT was normal (Figure 2(a)). Patient achieved progressive trilineage response by cycle 4. After cycle 11, she presented with shortness of breath, fatigability, and hypoxemia. Her auscultation revealed bibasilar crackles. A follow-up chest CT highlighted subpleural bilateral ground-glass opacities, subtle early honeycombing in posterior right lung base, and increased interstitial lung markings bilaterally (Figures 2(b) and 2(c)). In addition, increased bronchial wall thickness was observed (Figure 2(d)). Her blood cultures, urine legionella, and streptococcus antigens were negative. ESR and CRP were 63 mm/hr and 0.68 mg/dL, respectively. Her autoimmune panel showed ANA and p-ANCA antibodies positive at titers of 1 : 40 and 1 : 160. Her SCL-70 antibody was negative. Peripheral blood immunophenotypic analysis revealed normal absolute CD4+CD25^high^FOXP3+(Tregs), CD4+, and CD8+ T cells with reduced CD4 : CD8 ratio. Both CD4+ and CD8+ T cell compartments demonstrated an increase in activation state. Patient was initiated on prednisone at 20 mg orally daily. Her dyspnea, oxygen dependence, and pulmonary infiltrates gradually resolved after 8 weeks of treatment (Figure 2(e)). To date, 26 cycles of DAC have been delivered without additional complications.

## 4. Result

### 4.1. Patient Characteristics

As depicted in Table 1, median patient age was 71 years (range, 56–89). 5/7 (71%) of patients were males. All patients from the literature review were diagnosed with MDS and developed radiographic suggestion of BOOP/IPF while on azanucleoside. After initiation of respiratory symptoms, broad-spectrum antibiotics were started; however, an infectious etiology was not detected leading to progressive antibiotic deescalation. Symptoms were available in all patients with combination of fever, dyspnea, and/or dry cough observed in 5/7 (71%) of patients. In all cases, treatment with steroids and azanucleoside discontinuation resulted in quick symptoms improvement.

### 4.2. Characteristics of Pulmonary Complication

Symptoms were observed within a median of 1 cycle of treatment. Radiographically, diffuse bilateral interstitial infiltrates were observed in 5/7 (71%) of cases. As shown in Table 1, pathology was documented in 3/7 cases, revealing that diffuse alveolitis with honeycombing, bronchogenic granulomatous pattern, focal areas of intra-alveolar inflammation with necrosis, and fibrotic tissue were common findings in patients with available biopsies.

### 4.3. Characteristics of MDS

WHO 2008 classification was available in 5/7 cases representing RAEB-1 (2 cases), RAEB-2, MDS derived AML, and t-MDS, 1 case each (Table 1). Karyotypic abnormalities were reported in 4/7 patients including 3 patients harboring complex cytogenetic. At the time of symptoms onset, neutrophils, lymphocyte, and platelet counts were 690 (range, 90–4300/*μ*L), 895 (range, 490–1790/*μ*L), and 175000 (range, 12000–617000/*μ*L), respectively. For patients experiencing early onset of pulmonary complications, median ANC was 630 (range, 90–4300/*μ*L).

## 5. Discussion

Therapeutic alternatives for elderly patients with high-risk MDS and AML are limited with treatment success compromised by drug-induced complications. The incidence of azanucleoside induced pulmonary complications is unknown. Our report is the first in describing a late onset of azanucleoside induced lung injury, which contrasts with most of previously reported cases. A combination of fever, cough, and dyspnea should prompt detailed investigation, especially if no infectious etiology is detected. An important mechanism of lung injury associated with BOOP/IPF includes increased overexpression in neutrophils elastase leading to increased collagen content and fibrotic changes [9]. In our studied cohort, a low median ANC, described in most of patients with early onset manifestations, suggests direct drug tissue injury, rather than mediated by release of neutrophils inflammatory products. Widespread dysplastic hypogranular neutrophils would potentially render these cells incapable of functional degranulation during initial azanucleoside exposure. However, our late onset case, associated with successful hematologic improvement and normal Tregs, suggests disease remission and possible mechanism of lung injury resultant from robust immune reconstitution. Evidence of immune deregulation given CD4 memory skewing with reduced CD4/CD8 ratio, characteristic of activation states, might have facilitated permissible cue for BOOP/IPF. This is supported by suggestion of autoimmunity linked to her positive ANA and P-ANCA antibodies. A similar T cell activation profile is described in low-risk MDS patients with autoreactive damaging effector T cells responsive to immunosuppressive therapy [10]. BOOP/IPF originating after achievement of hematological response would be associated with gradual shift in lymphocyte responsiveness, increased T cell clonal frequencies. Similar to our case, Kotsianidis et al. suggested that azacytidine could induce autoimmune potentiation leading to sarcoidosis exacerbation in a patient with MDS [11]. The same authors reported that* in vivo* INF*γ* intracellular staining was significantly augmented after 28 days of azacytidine exposure. This observation highlights that azanucleosides pose chromatin configuration allowing increased cytokine overexpression that facilitates not only tumor control but also possible collateral normal tissue damage. In addition, it is possible that azacytidine promoted successful hematopoiesis resulting in functional neutrophils with potential for inducing lung parenchyma injury. Our patient favorable outcome suggests that azanucleoside induced antitumor immunity could result in enhanced vulnerability for late onset BOOP/IPF. Interestingly, Sekhri et al. demonstrated that, in a patient with ILD deriving from azacytidine treatment, transitioning from azacytidine to decitabine allowed not only therapy continuation but also resolution of ILD manifestations [6]. Despite authors suggest that lung injury could have resulted from drug-induced type I collage synthesis [6], it is also possible that distinct patterns of immune reconstitution for both drugs may account for this observation. Limitations of our study include lack of pathological confirmation of BOOP/IPF in our patient. Additionally, for the same patient, despite suggestion of deregulated immunity, a lack of functional T cell studies limits our ability to assign a direct cytotoxic role in lung tissue injury.

In summary, our study suggests a time-dependent presentation for BOOP/IPF in the context of azanucleosides therapy, which could be consistent with 2 distinct underlying mechanisms. Careful evaluation of patients presenting with noninfectious febrile respiratory symptoms during treatment with azanucleosides should include investigation for potential BOOP/IPF. Mechanisms of lung injury linked to epigenetic therapy should be investigated, especially in the context of potential uncharacterized immune and epigenetic mechanisms.

## Figures and Tables

**Table 1:** Clinical and laboratory characteristics of MDS and AML patients with pulmonary complications treated with azanucleosides therapy.

Case	Age/sex	Symptoms	Cycles of AZA	Initiation of symptoms (days)	Radiographic findings	Lung pathology	CRP^a^ (mg/dL)	MDS WHO 2008 classification	Cytogenetic	R-IPSS	ANC^b^ (cells/*μ*L)	ALC^c^ (cells/*μ*L)	Platelet count (/*μ*L)	Ref.
1	72/M	Fever, shortness of breath	1	3	Interstitial pneumonitis, ground-glass opacities	NA	10.2	RAEB-1	46, XY, der(1;7)(q10;p1) [17/20]	Very high	90	540	18000	[3]

2	64/M	Dry cough, fever, and chills	2	2	Left lower lobe infiltrate	Fibrinous and organizing pneumonia	NA	t-MDS^d^	NA	NA	140	490	12000	[4]

3	74/M	Dry cough, shortness of breath	1	7	Nonsegmental consolidation/ground-glass opacities	NA	1.25	RAEB-1^e^	Complex	Very high	630	1790	17000	[5]

4	56/M	Dry cough, fever	1	2	Nodular opacities, bilateral airspace disease	Interstitial lung disease, organizing pneumonia with bronchocentric granulomatous pattern	NA	RAEB-2	NA	NA	750	NA	12000	[6]

5	71/M	Fever, shortness of breath	1	14	Diffuse bilateral interstitial/alveolar infiltrates	Focal areas of intra-alveolar acute inflammation and necrosis	NA	NA	NA	NA	NA	NA	NA	[7]

6	74/F	Fever, dry cough, and shortness of breath	2	5	Reticulonodular and ground-glass shadowing and small pleural effusions	NA	NA	NA	Complex	Very high	4300	NA	342000	[8]

7	Case	Shortness of breath	11	330	Bilateral interstitial lung infiltrates	NA	0.68	MDS derived AML	Complex^*∗*^	Very high	1750	1250	579000	Case

^a^CRP: C-reactive protein; ^b^ANC: absolute neutrophil count; ^c^ALC: absolute lymphocyte count; NA: not available; ^d^t-MDS: treatment-related MDS; ^e^RAEB: refractory anemia excess blast; ^*∗*^karyotype included 10 chromosomal abnormalities.
